# Using Physical Modeling to Optimize the Aluminium Refining Process

**DOI:** 10.3390/ma15207385

**Published:** 2022-10-21

**Authors:** Tomáš Prášil, Ladislav Socha, Karel Gryc, Jana Svizelová, Mariola Saternus, Tomasz Merder, Jacek Pieprzyca, Martin Gráf

**Affiliations:** 1 MOTOR JIKOV Slévárna a.s., Kněžskodvorská 2277, 370 04 České Budějovice, Czech Republic; 2 Faculty of Mechanical Engineering, University of West Bohemia, Univerzitní 2732, 301 00 Plzeň, Czech Republic; 3 Environmental Research Department, Institute of Technology and Business in České Budějovice, Okružní 517/10, 370 01 České Budějovice, Czech Republic; 4 Faculty of Materials Engineering, Silesian University of Technology, Krasinskiego 8, 40-019 Katowice, Poland

**Keywords:** aluminium, refining, rotary impeller, physical modeling

## Abstract

Concern for the environment and rational management of resources requires the development of recoverable methods of obtaining metallic materials. This also applies to the production of aluminium and its alloys. The quality requirements of the market drive aluminium producers to use effective refining methods, and one of the most commonly used is blowing an inert gas into liquid aluminium via a rotating impeller. The efficiency and cost of this treatment depends largely on the application of the correct ratios between the basic parameters of the process, which are the flow rate of the inert gas, the speed of the rotor and the duration of the process. Determining these ratios in production conditions is expensive and difficult. This article presents the results of research aimed at determining the optimal ratio of the inert gas flow rate to the rotary impeller speed, using physical modeling techniques for the rotor as used in industrial conditions. The tests were carried out for rotary impeller speeds from 150 to 550 rpm and gas flow rates of 12, 17 and 22 dm^3^/min. The research was carried out on a 1:1 scale physical model, and the results, in the form of visualization of the degree of gas-bubble dispersion, were assessed on the basis of the five typical dispersion patterns. The removal of oxygen from water was carried out analogously to the process of removing hydrogen from aluminium. The curves of the rate of oxygen removal from the model liquid were determined, showing the course of oxygen reduction during refining with the same inert gas flows and rotor speeds mentioned above.

## 1. Introduction

A common method of refining liquid aluminium is blowing refining gas into a metal bath [1,2,3,4]. It consists of blowing a certain value of an inert gas stream (usually argon or a mixture of argon and nitrogen) into the metal bath through a rotating rotor. The main purpose of this treatment is to remove gases contained in the bath, especially hydrogen, which lowers the mechanical properties of finished aluminium products [5,6,7]. Additionally, metallic and non-metallic impurities that arise mainly during secondary aluminium smelting are also removed [8,9].

Physical phenomena occurring during the refining of aluminium and its alloys in refining reactors using inert gas injection are very complex [10,11]. When the rotor rotates at a high speed, the bubbles break up under the shear force acting on them at the rotor edge [7]. In vigorously agitated reactors, the behavior and mechanism of gas bubbles, as well as the hydrodynamic field, are complicated. The hydrodynamic field is characterized by turbulence and an uneven rate of energy dissipation and the presence of vortices moving in different directions [12,13,14,15]. This means that the bubbles in such systems are subjected to various internal and external forces that lead to their breakage [16,17]. The size of gas bubbles is influenced most by rotating speed, stirring power, surface tension and liquid density. Most experimental studies [18,19,20,21,22] that consider bubble or droplet rupture in vigorously stirred reactors have attempted to characterize this phenomenon by examining the size distribution of the bubbles or the maximum diameter of the derived bubbles, i.e., “daughter” bubbles. Additionally, as bubbles are generated by the rotor, they may encounter turbulent vortices relatively close to the rotor and, thus, immediately break up into several smaller daughter bubbles. The number of broken bubbles further away from the impeller is rather limited [17,20,22]. About 90% of the introduced bubbles are found to approach the rotor and then deflect towards the wall [21]. Therefore, the rotor geometry is very important.

The effectiveness of the process depends on many factors. The main process parameters influencing its efficiency are the rotor speed and the inert gas flow rate [22,23,24]. These parameters directly affect the mechanism of gas dispersion in the volume of the refined metal bath and the nature of the movement of the liquid metal in the refining vessel [25,26]. For refining reactors with a rotor, Oldshue [27] described four degrees of gas-bubble scattering in liquid metal: column flow, or the formation of geysers on the surface of the liquid; minimum dispersion; fine dispersion and uniform dispersion. After analyzing this research, one more case was added to these four schemes [28] during which turbulence and chain flow arose, which is a highly undesirable effect.

With a given rotor design, the required inert gas dispersion is achieved by a reasonable compromise between the intensity of the gas blown in and the rotor speed, while maintaining minimum undulations of the liquid metal surface [29,30,31,32]. The correct selection of these parameters is possible mainly experimentally [33,34,35,36,37,38]. However, conducting this type of research in industrial conditions is very difficult and expensive for fundamental reasons.

One of the tools often used to define the basic process parameters and to understand the phenomena occurring in the process is physical modeling [23,24,25]. More and more often, this modeling is supplemented and verified by numerical modeling, which can be used to assess the phenomena occurring during the injection of inert gas into liquid aluminium. This article uses physical modeling to evaluate the operation of a rotor used in an industrial plant. The aim of these studies was to optimize the refining process and, thus, to improve its economy. This way, the knowledge state is expanded by direct operational application. The novelty consists of a complex method of assessment using several methods and bringing theoretical knowledge in line with technical practice.

## 2. Construction of Model Stand

The construction of physical models of metallurgical reactors, where the studied phenomena take place in liquid metal, is usually based on the equations of fluid mechanics. The construction of these models must meet certain conditions so that the results correspond to the phenomena occurring in industrial devices. These conditions are obtained by applying theorems written using the theory of similarity. The main problem is then to determine the dynamic similarity on the basis of which it is possible to determine the kinematic similarity. This similarity involves the maintenance of constant proportions between the forces occurring in the model and the forces acting in the real object. A commonly used method to ensure dynamic similarity is a dimensional analysis of the mathematical description of the phenomenon under study [39,40]. When considering the interaction of forces in fluids, the Navier–Stokes equations are used. The criteria numbers can be determined on the basis of the N–S equations, using the π law (Buckingham π theorem) for the metallurgical reactor.

The similarity of the values of the criteria numbers in the model and in the real object ensures that the condition of dynamic similarity is met. In general, it is not possible to meet all the criterion numbers in the model. However, sufficiently satisfactory modeling results can be obtained using so-called incomplete dynamic similarity, in which the compliance of the value of one (for uncomplicated flows) or several criteria numbers (characterizing a phenomenon significant from the research point of view) are met. In most cases, when considering single-phase flows, the sufficient criterion for similarity is equal to the Froude number. When considering the aluminium refining process, the gas flow in the reactor must be taken into account. In multiphase flows, it is necessary to use a modified Froude number-considering the differences in density between the liquid (metal) and the gas (inert gas)-and the Weber number (*We*) in order to account for surface phenomena. The modified Froude (*Fr_N_*) number then takes the form:

(1) $$Fr_{N}=\frac{\rho_{g}\cdot v^{2}}{\rho_{l}\cdot g\cdot L}$$

where *ρ_g_* is gas density; *ρ_l_* is liquid density; *g* is acceleration due to gravity; *L* is liquid height in the model and *v* is gas injection velocity.

The Weber number expresses the ratio of inertial forces to surface tension forces. In a liquid, bubbles can remain stable below a given size. For a rotor gas injection case, important is the ratio between the inertial field force, F_c_, and the surface tension force, F_s_. If the surface tension dominates, then F_c_/F_s_ < 1.0. When the ratio becomes sufficiently larger than 1.0, the bubbles will break into smaller bubbles. Assuming that the break-up process is fast and only takes place close to the rotor side-holes, the Weber number can be used to decide whether the breakup will occur. The Weber (*We*) number has the following form:

(2) $$We=\frac{v^{2}\cdot L\cdot \rho }{\sigma }$$

where *ρ* is density; *σ* is surface tension; *L* is liquid height in the model and *v* is gas injection velocity.

**Figure 1:**
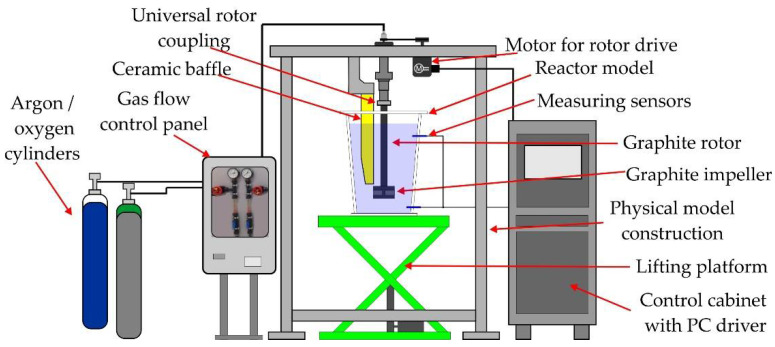
Schematic diagram of the FDU physical (water) model.

The model test stand meets the requirements of the segmented physical model of the FDU unit. The main segment in the form of a refining ladle model is made of transparent materials at a scale of 1:1. Water was used as the medium representing liquid aluminium. The hydrogen dissolved in the liquid aluminium is represented by oxygen that saturates the water. Argon is the refining gas. A schematic diagram of the physical model of the FDU is shown in Figure 1.

The basic structural elements of the model are: a pantographic lifting platform for precise positioning of the refining vessel model, a 1:1 scale refining ladle model, a supporting structure (including a rotor drive motor, universal rotor clutch, graphite rotor, ceramic baffle mounting, dispenser of gas to the rotor and laser tachometer), a gas-flow control panel (oxygen → gasification, argon → refining), two probes for measuring the oxygen content in water and a control panel (cabinet equipped with a computer controlling the rotor speed and a recorder of the results of the degassing process).

**Figure 2:**
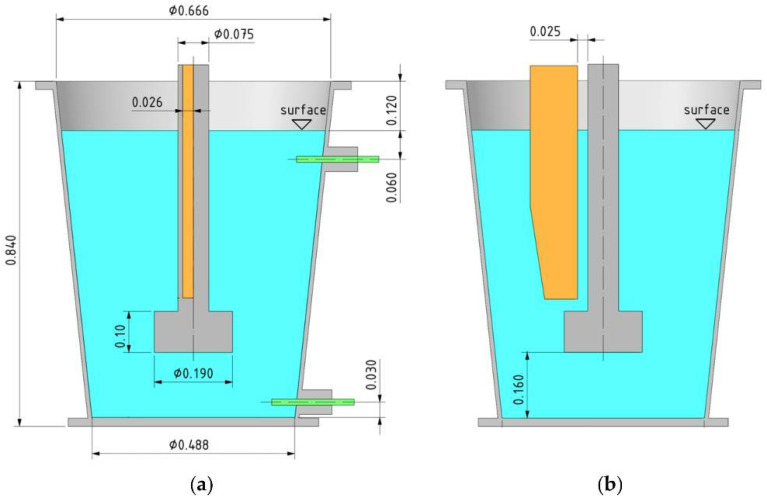
Basic dimensional data (m) of the physical model and individual components: (**a**) front view, (**b**) side view.

The entire load-bearing structure of the physical model ensures stability and safety during the experiments. The design of the lifting platform enables smooth adjustment of the rotor immersion depth in the ladle models. The model is equipped with a control and recording panel equipped with numerous electronic devices which enable precise gas distribution to the ladle model and real-time recording of its flow value. The diagram and basic dimensions of the refining ladle model are shown in Figure 2.

During the design of the test stand, it was assumed that an analogue of the removal of dissolved hydrogen from molten aluminium in industrial conditions would be the removal of dissolved oxygen from the model liquid (water) using an argon stream. Therefore, InPro6860i/12/120/mA Ex optical probes (METTLER-TOLEDO, USA) were installed in the model for continuous measurement of the oxygen content in the model liquid, enabling measurement in the expected range of oxygen concentration up to 26 mg∙L^−1^ (26 ppm). It was necessary to develop a system for the probes that would meet the requirements for the measurement of the oxygen concentration, including communication via a PC, data archiving and evaluation. The system itself consists of a stabilized 24 V power supply and our own hardware converter with software for monitoring and recording the values, which was installed on a connected PC.

## 3. Research Methodology

Laboratory experiments focused on physical modeling under standard operating conditions, obtaining information about the course of the refining processes and the influence of important process variables on its efficiency in the FDU. Table 1 shows the operating parameters of an industrial reactor and the physical model.

**Figure 3:**
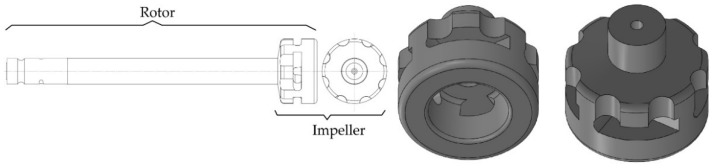
A 3D view of the rotor (impeller).

The research covered the structure of the rotor (rotor shaft and impeller) used in industrial conditions, shown in Figure 3.

The methodology of the research was as follows: setting the ladle model in the required position for the rotor, i.e., 0.16 m from the bottom of the ladle model; filling the ladle model with water to the required level of 0.72 m from its upper edge; setting the required gas flow while maintaining the gas pressure of 0.4 bar; saturation of the model liquid with oxygen at a rotor speed of 350 rpm. This was then followed by a period of homogenization of the oxygen concentration in the water volume and stabilization of the system. During this period, the rotor is turned off and the oxygen concentration reaches the required value, the required speed for a given variant of the experiment is set and measurement starts. The experiment ends when the average oxygen-concentration value reaches 0.5 ppm, then the impeller rotation is turned off and the gas supply is cut off.

In order to determine the optimal ratio of the value of the intensity of the blown gas to the value of the rotor speed, the experiments were carried out in a series of at least 3 experiments for 15 different variants of the processing parameters of the refining treatment. The research plan, therefore, included 45 experiments using the water model stand for refining liquid aluminium or its alloys. The variants of the experimental parameter settings are presented in Table 2.

## 4. Research Results

The results have two forms. The first is the visualization of the refining process, which document the fluid flow and the distribution of argon bubbles in each variant well. The second is the curves of the oxygen removal rate from the model liquid, showing the course of the reduction in oxygen concentration during refining with different inert gas flows and rotor speeds.

### 4.1. Results of Visualization

**Figure 4:**
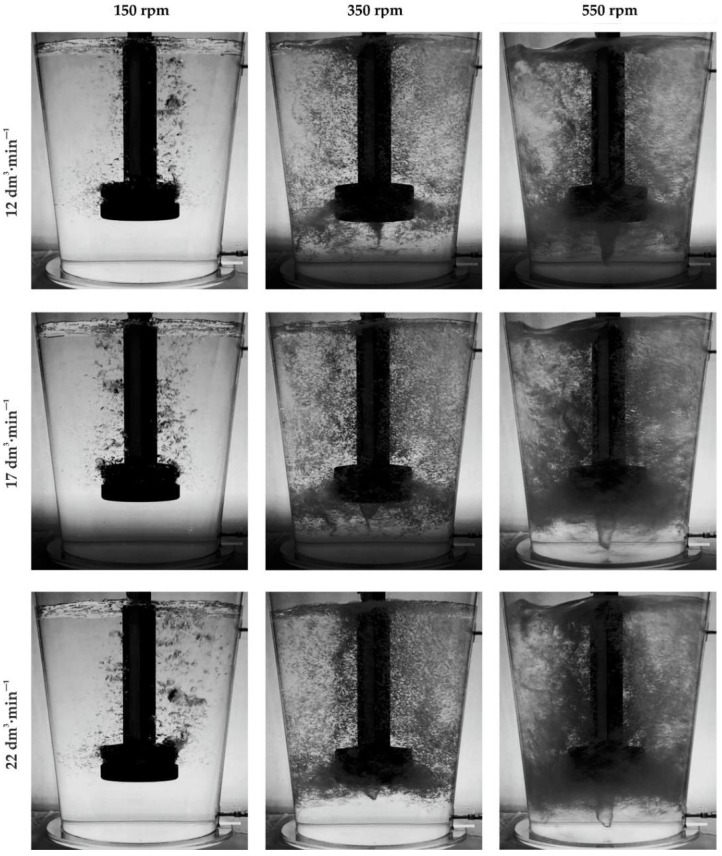
Demonstration of the nature of internal flow, behavior and distribution of emerging bubbles of blown argon.

Sample images showing the nature of the internal flow, behavior and distribution of the argon bubbles appearing in the argon flow from 12 dm^3^·min^−1^ to 22 dm^3^·min^−1^ are shown in Figure 4.

**Figure 5:**
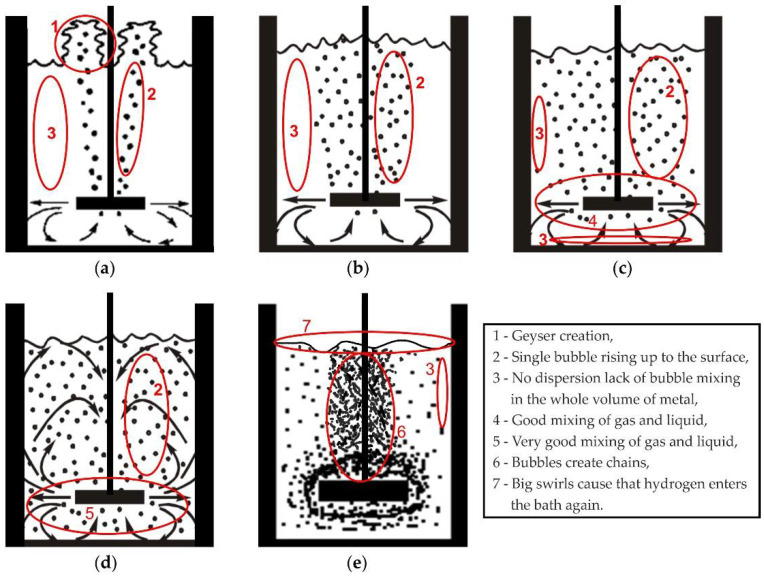
Typical types of inert gas dispersion in liquid aluminium formed during the refining operation: (**a**) no dispersion (geyser and single bubbles creation), (**b**) minimal dispersion, (**c**) fine dispersion, (**d**) uniform dispersion, (**e**) excessive dispersion (chain flow and swirls).

During the analysis of the results of the visualization tests, the degree of gas-bubble dispersion in the model liquid and the surface condition of the model liquid in the refining ladle were assessed from the point of view of the formation of strong turbulent zones in the area of its contact with the brake baffle. These two phenomena sufficiently characterize the required course of the process from the physical point of view. The following method was adopted as the basic criteria for assessing the degree of gas-bubble dispersion in the model liquid: no dispersion (geyser and single bubbles creation), minimal dispersion, fine dispersion, uniform dispersion and excessive dispersion (chain flow and swirls). Figure 5 shows diagrams of all of these types of dispersion along with their characteristic features. Uniform dispersion is the most desirable type.

On the basis of the dispersion diagrams and analysis of the photographs, all variants of the experiments carried out in Table 3 are listed as a specific type of dispersion.

**Figure 6:**
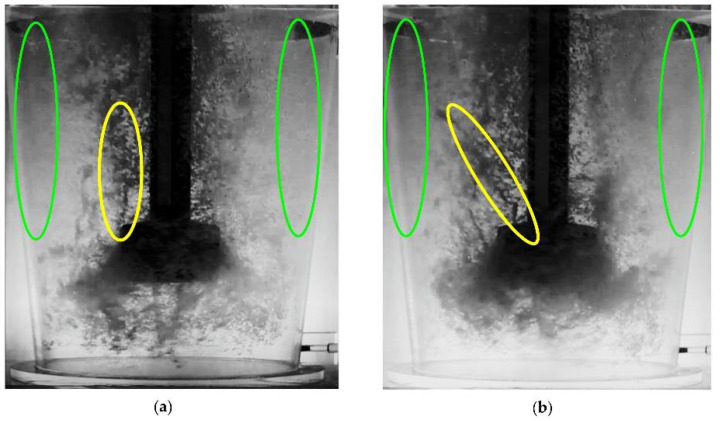
Characteristic features of excessive dispersion (creation of swirls in yellow and dead zones–no dispersion in green) for different variants of experiments: (**a**) 550 rpm, 12 dm^3^·min^−1^ (**b**) 550 rpm, 17 dm^3^·min^−1^.

On the basis of the visualization, it was found that the most desirable type of dispersion, i.e., close to uniform dispersion, is achieved for the blown-gas stream value above 17 dm^3^·min^−1^. This effect occurs at rotor speeds of 350 rpm. At the same time, for the parameters 22 dm^3^·min^−1^ and 550 rpm, a tendency towards excessive dispersion is observed. Excessive dispersion is observed at 550 rpm in almost all of the tested variants of gas flow rate. Excessive dispersion is characterized by the creation of swirls which are marked in yellow in Figure 6, and in some places, there is a lack of dispersion, marked in green in Figure 6. On the other hand, with a rotor speed of 450 rpm, a dispersion similar to the uniform type is possible when the gas stream value is greater than 22 dm^3^·min^−1^ (see Table 3, variant 12).

The analysis of the behavior of the surface of the model liquid during the experiment revealed a tendency towards excessive undulations in cases when the gas dispersion becomes excessive (see Figure 4 in all cases at 550 rpm).

### 4.2. Oxygen-Removal-Rate Curves

**Figure 7:**
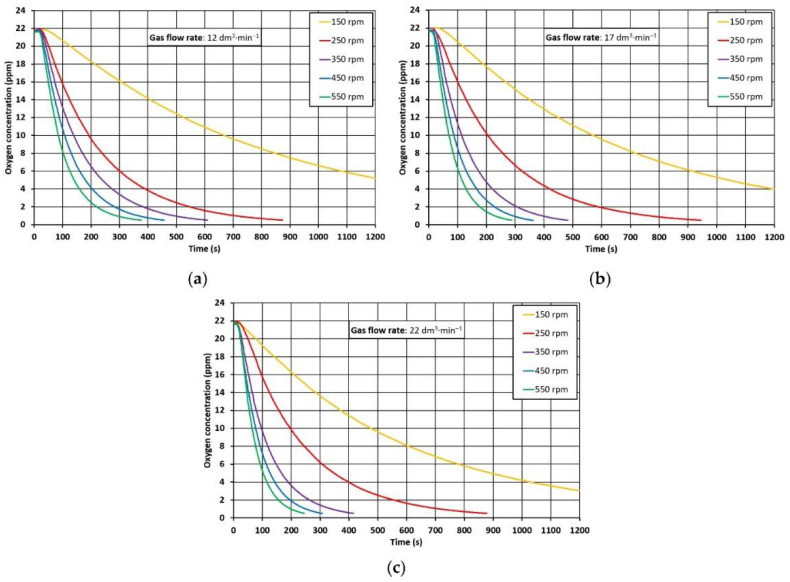
Demonstration of concentration curves measured on a physical model. Gas flow rate: (**a**) 12 dm^3^·min^−1^, (**b**) 17 dm^3^·min^−1^, (**c**) 22 dm^3^·min^−1^.

Oxygen-removal-rate curves were derived from measurements made during the experiments with METTLER-TOLEDO InPro6860i/12/120/mA Ex optical probes. Examples of the results of these measurements in the form of the dependence of the change in oxygen concentration in the model liquid as a function of time are shown in Figure 7.

**Figure 8:**
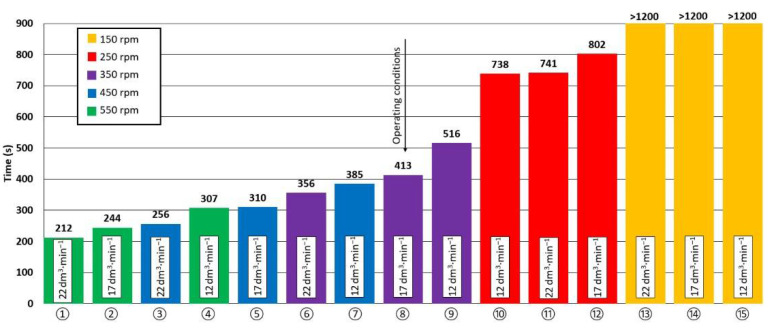
The minimum times of oxygen removal from the model liquid to the content of 1 ppm.

The quantification of the problem in the form of a graph illustrating the effectiveness of the liquid-aluminium refining process is shown in Figure 8. The minimum process duration needed to obtain the oxygen concentration in the model liquid lower than or equal to 1 ppm was adopted as the criterion for assessing the effectiveness of the refining process. Assuming that the duration of the refining process is a function of the oxygen concentration in the model liquid $T_{R}\left(C_{O_{2}}\right)$, the minimum refining time is achieved at $C_{O_{2}}\;$ ≤ 1 ppm.

It is evident from the graph in Figure 8 that, with a few exceptions, shorter oxygen-concentration reduction times to 1 ppm were achieved at higher argon flow rates and rotor speed. The shortest oxygen-concentration reduction time was achieved in variant 1 with the rotor speed of 550 rpm and with the argon flow of 22 dm^3^·min^−1^. On the other hand, the variants with the rotation of 150 rpm showed the longest reduction times of oxygen concentration in the model liquid. For these variants, the target oxygen concentration of 1 ppm was not reached within the measurement time of 1200 s. Figure 8 also shows that some variants achieve similar oxygen-concentration reduction times at lower rotor speeds and higher argon flows (e.g., variant 6, variant 7). This fact is important when considering the negative influence of higher speeds on the undulation of the metal surface (increase in the phase boundary), which is disadvantageous due to the possibility of re-oxidation and introduction of impurities into the volume of the alloy from its surface.

Variant 8, which is currently used in operational conditions (350 rpm, 17 dm^3^·min^−1^), was also modeled, and the oxygen-concentration reduction time was 413 s. Figure 8 shows the possibility of intensifying the process by changing one process parameter. For example, if the argon flow is maintained at 17 dm^3^·min^−1^ and the speed is increased to 450 rpm, the time for decreasing the oxygen concentration is shortened by approximately 100 s (variant 5). This change may also have a positive effect on the reduction in the refining time under operating conditions.

## 5. Conclusions

The following conclusions can be drawn based on the research:

- The efficiency of refining liquid Al using a rotor reactor depends mainly on the correct distribution of gas-bubble dispersion in the bath volume and the intensity of the metal mixing. The required formation of the physical phenomena is influenced by the proper selection of the process parameters, which are the intensity of the blown gas and the rotor speed.
- The required course of the refining of liquid Al can be achieved through a reasonable compromise between the values of the technological parameters. With the increase in the rotor speed, the fragmentation and the number of gas bubbles in the bath increase, as well as the risk of creating excessive waves on its surface.
- The rotor speed has a dominant influence on the movement of the surface of the liquid Al. Centrifugal force breaks the stream of gas into small bubbles moving in the bath perpendicular to the axis of the refining vessel. This movement has a positive effect on limiting the undulations of the bath surface.
- The selection of the process parameters should, therefore, be carried out so that with the minimum possible rotor speed, the rate of the blown-gas stream ensures uniform dispersion of its bubbles in the metal bath.
- Uniform dispersion was obtained with a rotor speed of at least 350 rpm for the tested rotor structure and the volume of the refining ladle, but this value should not exceed 450 rpm. At a rotor speed of 550 rpm, turbulence and chain flow are observed at each rate of gas flow, leading to excessive dispersion. Excessive dispersion is a case of the formation of swirls or vortices, or even small geysers, on the surface of a liquid metal. From the point of view of the process, this is not advantageous, as it may lead to the reintroduction of hydrogen into the aluminum and its alloy and the appearance of porosity in the alloy and, thus, a reduction in its mechanical and strength properties.
- As for the gas flow rate, the most economical is 17 dm^3^·min^−1^. Good results can also be obtained with 22 dm^3^·min^−1^, but for industrial conditions, it is not advisable. Rather, the rotor speed should be increased to 400 or 450 rpm. This could shorten the refining time by about 25%, which is highly recommended for industrial use.
- Another solution that should be taken into account in subsequent tests is the height of the rotor. The rotor could be lowered, and then in some cases no dead zones would be formed under the rotor (without dispersion).

## Figures and Tables

**Table 1:** Standard FDU operating parameters.

Parameters	Industry	Physical Model	Unit
	Value		
Liquid aluminium/water-reactor capacity	0.074		m^−3^
Reactor height	0.840		m
Liquid working level	0.720		m
Scale	1:1		-
Material for rotor (impeller)	Graphite		-
Amount of baffle	1		-
Inert gas	Nitrogen	Argon	-
Liquid	Aluminium	Water	-
Temperature	993	293	K
Density	2345	998	kg·m^−3^
Dynamic viscosity	1005	1000	Pa·s
Surface tension	0.868	0.072	N·m^−1^
Froude’s number	0.121	0.121	-
Weber number	133.35	595.4	

**Table 2:** Process parameters used in modeling research.

Parameters	Value														
Variants of experiments	1	2	3	4	5	6	7	8	9	10	11	12	13	14	15
Rotor speed, rpm	150			250			350			450			550		
Gas flow rate, dm^3^·min^−1^	12	17	22	12	17	22	12	17	22	12	17	22	12	17	22

**Table 3:** Summary of visualization results—different types of gas-bubble dispersion.

Parameters	Mark														
Variants of experiments	1	2	3	4	5	6	7	8	9	10	11	12	13	14	15
Type of dispersion	ND	MD	MD	ND	MD	MD	FD	UD	FD	FD	FD	UD	ED	ED	ED

ND—no dispersion; MD—minimal dispersion, UD—uniform dispersion; ED—excessive dispersion; FD—fine dispersion.

## Data Availability

Not applicable.
